# Supplementary material for: Copper (II) Species with Improved Anti-Melanoma and Antibacterial Activity by Inclusion in β-Cyclodextrin
Source: Int J Mol Sci. 2023 Jan 31;24(3):2688. doi: 10.3390/ijms24032688 (PMC9916925; doi:10.3390/ijms24032688)
Supplement: Supplementary file 1 [file ijms-24-02688-s001.zip › ijms-2153883-supplementary.pdf]

International Journal of *Molecular Sciences*

MDPI

Article

Copper (II) species with improved anti-melanoma and antibacterial activity by inclusion in β–cyclodextrin

Alina Tirsoaga [1], Victor Cojocaru [2], Mihaela Badea [2], Irinel Adriana Badea [1,*], Arpad Mihai Rostas [3], Roberta Stoica[4], Mihaela Bacalum [4], Mariana Carmen Chifiriuc [5,6,7], and Rodica Olar [12*]

[1] University of Bucharest, Faculty of Chemistry, Department of Analytical and Physical Chemistry, 4-12 Regina Elisabeta Av., District 3, 030018 Bucharest, Bucharest, Romania
[2] University of Bucharest, Faculty of Chemistry, Department of Inorganic and Organic Chemistry, Biochemistry and Catalysis, 90–92 Panduri Str., District 5, 050663 Bucharest, Romania
[3] National Institute for Research and Development of Isotopic and Molecular Technologies, Department of Physics of nanostructured systems, 67-103 Donat Str., 400293 Cluj-Napoca, Romania
[4] Horia Hulubei National Institute for Physics and Nuclear Engineering, Department of Life and Environmental Physics, 30 Reactorului Str., 077125 Magurele-Ilfov, Romania
[5] Department of Microbiology, Faculty of Biology, University of Bucharest, 1-3 Aleea Portocalelor Str., District 5, 060101 Bucharest, Romania
[6] Romanian Academy of Scientists, 54 Spl. Independent,ei Str., District 5, 050085 Bucharest, Romania
[7] The Romanian Academy, 25, Calea Victoriei, Sector 1, District 1, 010071 Bucharest, Romania
* Correspondence: irinel.badea@chimie.unibuc.ro (I.B.); rodica.olar@chimie.unibuc.ro (R.O.)

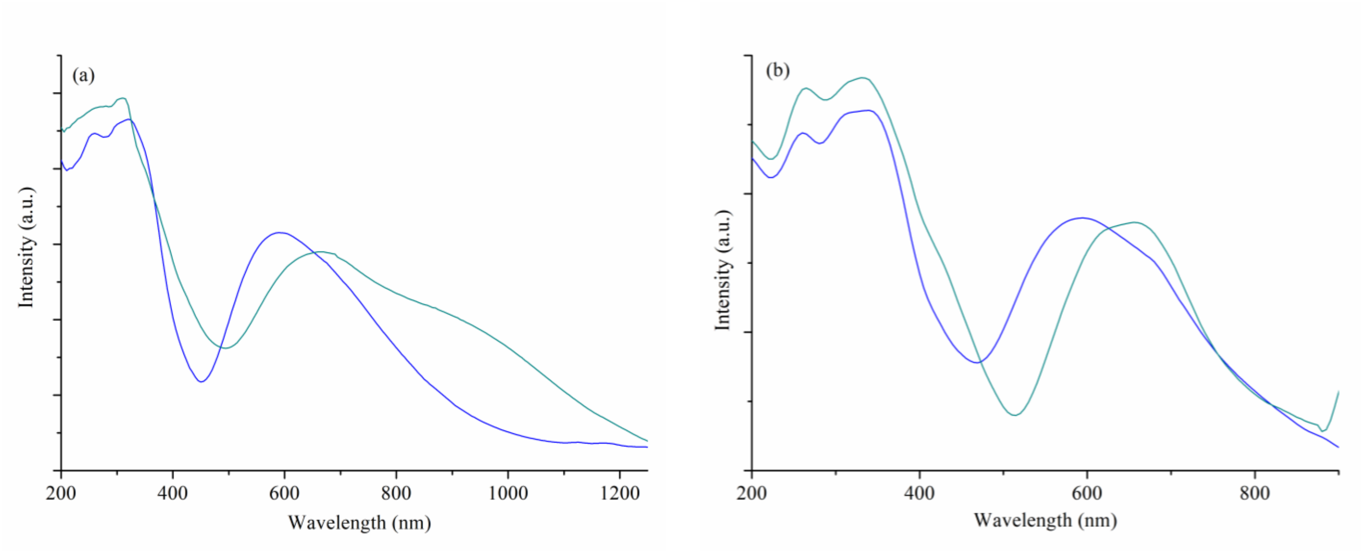

Figure S1. Solid state UV-Vis spectra of complexes (**1**) (dark blue) and (**1a**)@β-CD (dark green) (a) and (**2**) (dark blue) and (**2a**)@β-CD (dark green) (b) (diffuse reflectance technique with spectralon as reference).

Table S1. Absorption maxima (cm^{-1}) in IR spectra of β-CD, (**1a**)@β-CD, and (**2a**)@β-CD and their assignements.

β-CD	(1a)@β-CD	(2a)@β-CD	Assignements
3525 vs, 3480 vs, 3421 vs, 3340 vs, 3266 vs, 3222 vs	3405 m, wide	3386 s, wide	ν(OH)
-	3072 m	3050 m	ν(CH)aromatic
2927 m	2926 w	2926 m	ν(CH)
1645 m	1629 s	1629 s	δ(OH)
-	1551 s	1552 s	ν(C=N)
1155 s	1143m	1120 s	ν(C-O-C)
1054 s	1080 m	1080 s	ν(C-OH)
-	1089 vs	1079 vs	$\nu_3(ClO_4)$
-	781 w, 724 w	778 m, 731 w	γ(CH)aromatic
-	624 m	624 m	$\nu_4(ClO_4)$
-	485 w	485 w	ν(Cu-O)
-	438 w	430 w	ν(Cu-N)

vs = very strong, s = strong, m = medium, w -weak.
